# Enhancing Robotic-Based Propeller Blade Sharpening Efficiency with a Laser-Vision Sensor and a Force Compliance Mechanism

**DOI:** 10.3390/s23115320

**Published:** 2023-06-03

**Authors:** Yong-Sheng Cheng, Syed Humayoon Shah, Shih-Hsiang Yen, Anton Royanto Ahmad, Chyi-Yeu Lin

**Affiliations:** 1 Department of Mechanical Engineering, National Taiwan University of Science and Technology, Taipei 106, Taiwan; cyongsheng213@gmail.com (Y.-S.C.); d10803823@mail.ntust.edu.tw (S.H.S.); u060112@hotmail.com (S.-H.Y.); anton.royanto@uika-bogor.ac.id (A.R.A.); 2 Center for Cyber-Physical System, National Taiwan University of Science and Technology, Taipei 106, Taiwan; 3 Taiwan Building Technology Center, National Taiwan University of Science and Technology, Taipei 106, Taiwan

**Keywords:** robotic grinding, vision sensor, compliance mechanism, compensation strategy, industrial application

## Abstract

The edge sharpness of a propeller blade plays a vital role in improving energy transmission efficiency and reducing the power required to propel the vehicle. However, producing finely sharpened edges through casting is challenging due to the risk of breakage. Additionally, the blade profile of the wax model can deform during drying, making it difficult to achieve the required edge thickness. To automate the sharpening process, we propose an intelligent system consisting of a six-DoF industrial robot and a laser-vision sensor. The system improves machining accuracy through an iterative grinding compensation strategy that eliminates material residuals based on profile data from the vision sensor. An indigenously designed compliance mechanism is employed to enhance the performance of robotic grinding which is actively controlled by an electronic proportional pressure regulator to adjust the contact force and position between the workpiece and abrasive belt. The system’s reliability and functionality are validated using three different workpiece models of four-blade propellers, achieving accurate and efficient machining within the required thickness tolerances. The proposed system provides a promising solution for finely sharpened propeller blade edges, addressing challenges associated with the earlier robotic-based grinding studies.

## 1. Introduction

Industrial robots are widely regarded as a potent asset in the advancement of industrial automation [1]. In recent years, there has been a growing interest in utilizing industrial robots as an effective tool for several automation applications to address a range of issues such as sustainability, labour management, energy efficiency, and worker safety in developed countries [2]. Industrial robots have the potential to outperform traditional CNC machines in machining complex workpieces due to their flexibility, affordability, and spacious work area [3]. In addition, the machining capabilities of industrial robots can be further enhanced by their adaptability to integrate a range of sensors and external mechanisms. This overcomes their relatively poor accuracy and stiffness, allowing them to achieve better performance in complex machining tasks [4,5,6,7,8,9]. In general, complex components can be characterized as free-form, thin-wall surfaces, complicated structures, and high-dimensional machining capability needs such as turbine blades in aerospace [10]. To attain better machining quality for complex components, the grinding process is often indispensable in ensuring accurate contouring and high-quality surface finish. Robot-assisted manufacturing has demonstrated remarkable capabilities in achieving high levels of accuracy and efficiency in grinding complex geometrical workpieces, a crucial requirement in numerous industrial sectors, especially automobile, aerospace, and shipbuilding [11].

A plethora of related work have been conducted in the field of robotic-based applications aimed at enhancing grinding accuracy through the implementation of diverse methodologies [12,13,14,15,16,17,18,19]. For instance, constant force control is a widely adopted technique in robotic grinding applications due to its ability to consistently achieve and ensure optimal performance [20,21,22]. Xu et al. [23] presented an active force control strategy for the robotic abrasive belt grinding of the aero-engine blades. A method of hybrid force–position is employed with PI/PD control, and incorporated a six-axis force/torque sensor at the end of the robot. To accomplish constant force during the grinding process, the force and position controls were used in the Z- and X-direction of the tool frame, respectively. However, despite the implementation of active constant force control, it is still challenging to guarantee the machining accuracy of the workpiece profile due to the potential deformation of the contact wheel in conformity. Therefore, to enhance the grinding outcome by analysing the impacts of the cut-in and cut-off paths of the blade workpiece, a cutting force model based on a force control mechanism was developed [24]. Lv et al. [25] investigated an alternative active force control method for grinding the aero-engine blade. However, the robotic belt grinding on the leading and trailing edges of a complex blade is widely considered a challenging task, primarily due to the changing curvature on the workpiece surface, which often leads in an uneven distribution of contact pressure. To overcome this issue, an adaptive trajectory planning method was proposed, which involves modifying the grinding trajectory by taking into account the contact state between the workpiece and the contact wheel. The improved robotic grinding trajectory was combined with constant force control to prevent the occurrence of over-grinding. Furthermore, Xu et al. [26] enhanced the force control methodology in robotic grinding by combining active force control [23] and passive force control with Kalmen filter information fusion in order to achieve better grinding results and stability. The passive force control is carried out by coupling a one-axis force sensor with a PID controller in the grinding equipment. The enhanced grinding stability is primarily attributed to the compliance characteristic of the passive control device, enabling it to absorb instant impact, dynamic energy, and mechanical vibration. In recent years, numerous studies have been conducted to investigate the efficacy of compliant force control in comparison to active constant force control, with the aim of improving the robustness of robotic grinding operations [27,28,29,30]. Wang et al. [31] designed a compliant force control-based end-effector for an industrial robot to suppress the mechanical vibration during the grinding process for large thin-wall workpieces. The results of their study demonstrate that the use of force control reduced vibration amplitudes by up to 75% in stable grinding conditions. Another study focused on the development of a self-designed passive compliance device, which was applied to external grinding equipment used for robotic belt grinding of superalloy blades resulting in reduced contact force fluctuations and improved surface profile accuracy and quality of the workpiece after grinding [32].

Although, employing active force control with a six-axis F/T transducer has been identified as an effective approach to achieve the desired outcome in robotic grinding [23]. However, its sustainability remains a challenge due to uncertain grinding conditions that may arise in various situations. Therefore, compliance force control is an alternative for stabilizing the grinding process regardless of hybrid active/passive control [26,29], external compliance mechanism [27,30], or compliant end-effector design [31,33]. According to the findings of Gonzalez et al. [34], the incorporation of compliance in the automated chamfering process was shown to be effective and stable. The experimental results revealed significant improvements in the machining performance, with compliance ensuring a uniform chamfer size during operation. Additionally, the researchers introduced a predictive model to analyse the machining process utilizing spring-based compliance tools.

On the other hand, it is hard to guarantee the machining accuracy for those complex workpieces which are easily deformed due to their large scale, low stiffness, and machining characteristics. Therefore, the robotic machining trajectory planned according to the standard CAD model may not be used directly to achieve the machining requirement. To address the issue of workpiece profile inconsistency, there is a feasible way by employing the vision sensor to identify the practical difference between the standard CAD model and the existing object [35,36,37]. Thus, the machining trajectory can be corrected or re-created in a real-time scenario according to the information acquired from the vision sensor. Xiao et al. [38] proposed a reverse compensation strategy in an adaptive trajectory planning method for grinding the blisk blades. The profile error is analysed in the point cloud model which is scanned by a blue-light scanner, then the machining trajectory is reverse compensated for according to the determined profile error vectors until the desired workpiece profile is reached. Yang et al. [39] presented a trajectory planning method combining reverse engineering and finite element mesh technology to create the robotic polishing trajectory in an on-site experiment scenario. The mesh model was obtained by mapping the finite element mesh arbitrary quadrilateral elements on the surface of the scanned point cloud, then the robotic polishing path was generated by sorting the index of mesh nodes. Li et al. [40] analysed the effects of a mismatch between the practically scanned model and the standard CAD model. The objective function considering the allowance weights is established to modify the robotic trajectory for stabilizing the grinding force. Rodriguez et al. [41] presented an automated robotic machining system that utilized a 3D-structured light scanner to tackle challenges such as geometrical distortions, part positioning, and edge location in the production of aero-engine casings. Despite the benefits of machine vision, it has limitations in eliminating errors associated with the workpiece. To enhance the accuracy of the finishing process, the complaint multi-edge solid tools and flexible abrasive tools were employed in the proposed automated cells for deburring and polishing. According to the mentioned literature, there is great potential to solve the inconsistency problem between the practical workpiece and the standard CAD model by analysing the profile error acquired from the vision sensors.

Recently introduced iterative compensation methods have revolutionized the machining capabilities of five-axis CNC machines, allowing them to excel in producing complex curved workpieces such as aero-engine blades [42]. However, the five-axis CNC machines may not be the most cost-effective option for applications involving intricate workpieces with complex geometries, such as jet ski propellers. This is due to the high establishment costs and manufacturing difficulties associated with these machines. In contrast, the casting and grinding processes can be a more cost-effective approach for producing propeller-shaped workpieces. However, the casting process can cause deformation in these types of workpieces, leading to shape inconsistencies that can make it challenging to achieve the required machining accuracy using the standard CAD model. This issue is especially pertinent for industries that rely heavily on precision manufacturing. To address the aforementioned issues, Cheng et al. [43], employed a laser-vision sensor to determine the profile data of the propeller blades at multiple specified positions. They implemented an iterative grinding compensation strategy to meet the machining requirement. Moreover, the longitudinal feed parameters were computed according to the residual material, and the robotic trajectory was adjusted using those feed parameters to grind the workpiece sub-areas that did not meet the machining requirement. However, long-term production of high-quality workpieces in robotic grinding applications is often impeded by factors such as varying contact pressure distribution, unpredictable abrasive wear, and structural deformation of the equipment, which can result in unstable machining efficiency.In order to overcome this challenge, this study proposes a compliance mechanism to enhance the performance and efficiency of robotic machining. A pneumatic cylinder is employed in the designed compliant mechanism and installed in a vertical-type grinding machine. The output pressure is actively regulated by an electronic proportional pressure regulator to enable effective grinding compensation. The machining efficiency is improved through the compliance mechanism by passively adjusting the piston rod position to compensate for the robotic trajectory when unpredictable profile errors of the workpiece exist. In addition, the mechanical vibration and dynamic impact can be suppressed during the robotic grinding process with the compliance mechanism applied, and structural deformation of grinding equipment is avoided to enhance the machining stability.

The rest of the paper is organized as follows: Section 2 presents the external compliant mechanism design and the improved grinding compensation strategy. Section 3 describes the setup of experimental architecture. Section 4 analyses the experimental process and result. Finally, Section 5 discusses the conclusion and future works.

## 2. Compliant Mechanism Design and Grinding Control Strategy

In variety of robotic grinding applications, stable and constant force control is a crucial factor that significantly impacts the quality and stability of the machining process and the workpiece. Extensive research has been conducted in the area of constant force control, exploring both active and passive control methods. The majority of active constant force control studies employ a closed-loop PID force-position control methodology and a six-axis force sensor mounted at the end of an industrial robot. However, this method requires processing and handling the force/torque data and transmitting the control signal in an extremely short time to adjust the robot’s motion and compensate for force and position errors. It is challenging to ensure stability in practical applications due to the grinding limitations in various situations.

In contrast, passive compliance force control typically focuses only on one-axis force control, aimed at absorbing direct impact and mechanical vibrations between the workpiece and the grinding equipment. In addition, passive compliance mechanisms offer an autonomous error compensation capability by adjusting the contact interface position. This is particularly useful in situations where a workpiece has a profile error that results in the failure of the planned standard robotic trajectory.

The impact of unpredictable abrasive belt wear, the complex relationship between grinding depth and contact force [18], and the workpiece deformation induced by casting processes are important considerations in the design of a compliant mechanism and improved grinding compensation strategy to enhance the performance of robotic grinding systems. This paper discusses the design and implementation of a compliant mechanism coupled with a laser-vision sensor to perform the iterative grinding compensation strategy to gradually eliminate the material residual of the workpiece. The proposed grinding strategy aims to achieve the required machining accuracy despite the presence of these challenges.

### 2.1. Compliance Mechanism Design

**Figure 1:**
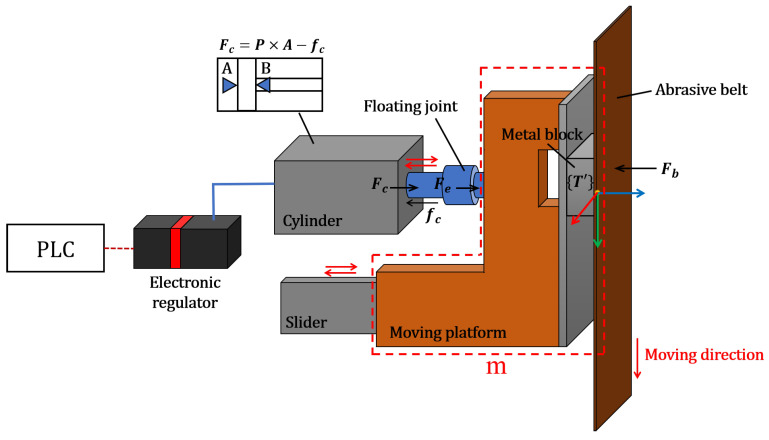
Design prototype of the active compliance mechanism.

Figure 1 showcases the prototype of the compliance mechanism design for the grinding process. The mechanism comprises a pneumatic cylinder, floating joint, linear slider, moving platform, and a metal block. The experimental workpieces used in the study are multi-blade jet ski propellers, which feature thin-thickness blades that are challenging to produce through casting. It is noteworthy that the edge thickness of the propeller blade is intentionally designed larger than the standard thickness. To achieve the required edge thickness, a substantial amount of redundant material must be removed using the grinding process. Hence, the metal block is chosen as the contact interface between the abrasive belt and the workpiece due to its ability to resist deformation during high-volume material removal. The conformity of the contact state can be neglected, but the dynamic impact and mechanical vibration issues still exist. Therefore, a pneumatic cylinder is utilized to enhance the machining stability by absorbing the effects generated during the grinding process.

In the compliant force control process, the output force $F_{c}$ is provided by a double-acting pneumatic cylinder according to the input pressure *P* controlled by the electronic regulator. $F_{c}$ can be calculated using Equation (1).

(1) $$F_{c}=P\times A-f_{c}$$

where *P* presents the pressure inside the pneumatic cylinder. The term *A* refers to the area in contact with the gas. $f_{c}$ is the friction force of the cylinder, which is usually considered as 3–20% of $F_{c}$. Therefore, Equation (1) can be rewritten as:

(2) $$F_{c}=\frac{\pi PD^{2}}{4}\left(1-\mu \right)$$

(3) $$F_{e}=cos\theta cos\phi F_{c}$$

where the term *D* is the piston diameter of the pneumatic cylinder and μ is the friction coefficient. The floating joint is installed between the piston rod and the moving platform to ensure smoothness of the stroke and eliminate the effects of assembly error. Thus, the slight pitch angle θ and deflection angle ϕ are considered for the real effective force $F_{e}$, as shown in Equation (3). Considering the entire operating system, the pneumatic cylinder continually suffers a tension force $F_{b}$ caused by the abrasive belt. Therefore, the piston rod can return stroke automatically by $F_{b}$ when low pressure is supplied to the pneumatic cylinder, enough to control the double-acting pneumatic cylinder by only using one air pressure source. To analyse the entire system’s force relationship for driving the pneumatic cylinder, Equations (4)–(6) are used to evaluate the required operating air pressure.

(4) $$F_{e}-F_{b}=\frac{\pi cos\theta cos\phi PD^{2}}{4}\left(1-\mu \right)-F_{b}=ma_{t}$$

(5) $$a_{t}=\frac{2S}{t^{2}}$$

(6) $$P=\frac{4F_{b}}{\pi cos\theta cos\phi D^{2}\left(1-\mu \right)}+m\frac{8S}{\pi cos\theta cos\phi D^{2}\left(1-\mu \right)t^{2}}$$

In Equation (4), the term *m* represents the mass of the moving platform which is marked by red dotted lines, as shown in Figure 1, whereas $a_{t}$ denotes the acceleration of the pneumatic cylinder for pushing the moving platform. According to Equation (5), $a_{t}$ can be computed by the stroke distance *S* and the operating time *t* of one stroke. The needed minimum *P* is computed according to Equation (6) by combining Equations (4) and (5).

(7) $$v_{max}=v_{0}+a_{t}\times t$$

(8) $$KE=\frac{1}{2}mv_{max}^{2}$$

Because $a_{t}$ is known from Equation (5), then the piston velocity $v_{max}$ of the outward stroke can be computed by Equation (7). The initial velocity $v_{0}$ can be considered as 0 since the piston is driven at the static state. The kinetic energy KE used to absorb the dynamic impact and mechanical vibration is found according to Equation (8). To apply real-time adjustment of the air pressure during the robotic grinding process, the gas consumption *Q* is considered by Equation (9).

(9) $$Q=nSA\frac{1.033+P}{1.033}\cdot 10^{-6}=nS\frac{\pi D^{2}}{4}\frac{1.033+P}{1.033}\cdot 10^{-6}$$

where *n* represents the stroke operating number within one minute. In addition, the term (1.033+P)/1.033 is the compression ratio between the outside atmospheric pressure and inside pneumatic cylinder pressure. Therefore, the suitable capability of the electronic regulator is calculated by considering the required overall data processing and communication.

### 2.2. Improved Iterative Grinding Compensation Strategy with the Compliance Mechanism

Achieving the required machining accuracy in the grinding process for workpieces that deviate significantly from the standard CAD model using the planned machining trajectory is challenging. In addition, the relationship between the abrasive belt and grinding depth is not predictable, which leads to challenges in sustaining the grinding stability and efficiency in high-production industries.Therefore, the improved grinding compensation strategy with a designed compliance mechanism is proposed to address the aforementioned issues.

**Figure 2:**
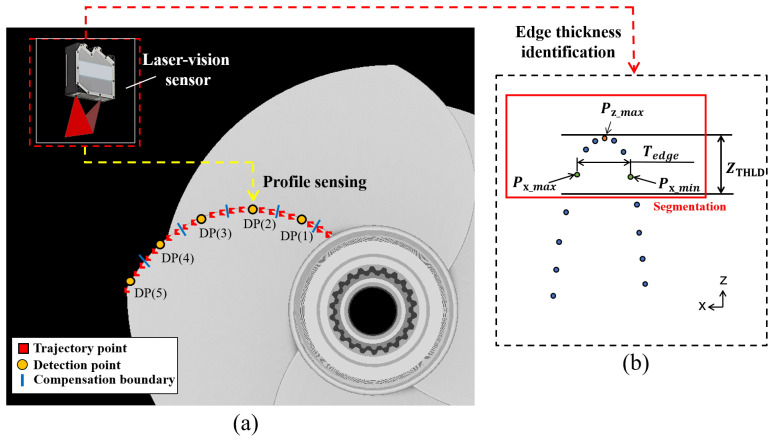
Schematic diagram of sensing and identifying the workpiece’s edge thickness through the laser-vision sensor. (**a**) Distribution of the detection points. (**b**) Process of determining the blade thickness.

The robotic grinding compensation strategy can be divided into two stages: (1) vision sensor detection and (2) grinding compensation. In order to conduct the grinding compensation processes for those workpiece sub-areas which have not met the machining requirement, the laser-vision sensor is employed to determine the practical workpiece’s material residual, as shown in Figure 2. The distribution of the trajectory points, detection points, and corresponding boundaries is shown in Figure 2a, and the edge thickness $T_{edge}$ can be computed in the profile data acquired from the laser-vision sensor, as shown in Figure 2b. The current workpiece’s edge thickness and material residual at each detection point are determined before applying the grinding compensation, then the operating pressure of the pneumatic cylinder for grinding each detection point’s corresponding area is evaluated according to the determined material residual.

(10) $$P_{next}=P_{low},if\left(T_{edge}<T_{std}\right)$$

(11) $$P_{\mathrm{next}\;}=P_{\mathrm{oper}\;},\;\mathrm{if}\;\left(T_{\mathrm{edge}\;}>T_{\mathrm{std}\;}\right),\;\mathrm{set}\;\left\{\begin{array}{c} P_{\mathrm{oper}\;}=P_{\mathrm{high}\;},\;\mathrm{if}\;\left(\nabla R>R_{THLD}\right)\\ P_{\mathrm{oper}\;}=P_{\mathrm{mid}\;},\;\mathrm{if}\;\left(\nabla R<R_{THLD}\right) \end{array}\right\}$$

(12) $$\nabla R=T_{edge}-T_{std}$$

where $P_{next}$ presents the operating pressure which is used in the grinding compensation process to adjust the output force of the pneumatic cylinder. The edge thickness $T_{edge}$ is computed from workpiece profile data acquired from the vision sensor, and then $T_{edge}$ is compared with the required machining thickness $T_{std}$ to find the material residual ∇R according to Equation (12). Furthermore, $P_{next}$ is set to low pressure $P_{low}$ making the output force $F_{c}$ smaller than the belt tension force $F_{b}$ when the detected $T_{edge}$ already satisfies the machining requirement; therefore, the grinding action is avoided during the robotic grinding compensation process due to the incomplete stroke of the pneumatic cylinder. Meanwhile, when the detected $T_{edge}$ is bigger than $T_{std}$, the $P_{next}$ is set to the operating pressure $P_{oper}$ providing enough $F_{c}$ to remove the material at the specified workpiece sub-area by driving the full stroke of the cylinder. The $P_{oper}$ can be set to high pressure $P_{high}$ or medium pressure $P_{mid}$ according to the value of ∇R. For instance, if the calculated ∇R is far over the material residual threshold $R_{THLD}$, then $P_{oper}$ is set to $P_{high}$ to remove more material volume. In contrast, if ∇R is less the $R_{THLD}$ but the $T_{edge}$ is still higher than $T_{std}$, $T_{edge}$ is close to $T_{std}$ but has not reached the required machining accuracy. Therefore, $P_{oper}$ is set to $P_{mid}$ making a full stroke of pneumatic cylinder using less output force to remove less material to avoid the over-grinding phenomenon.

**Figure 3:**
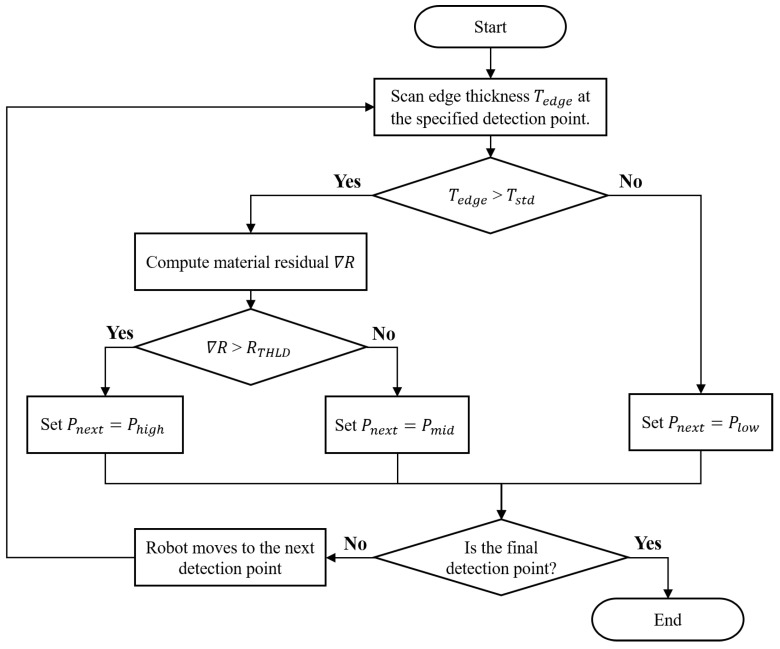
Flow chart of the vision sensor detection process.

**Figure 4:**
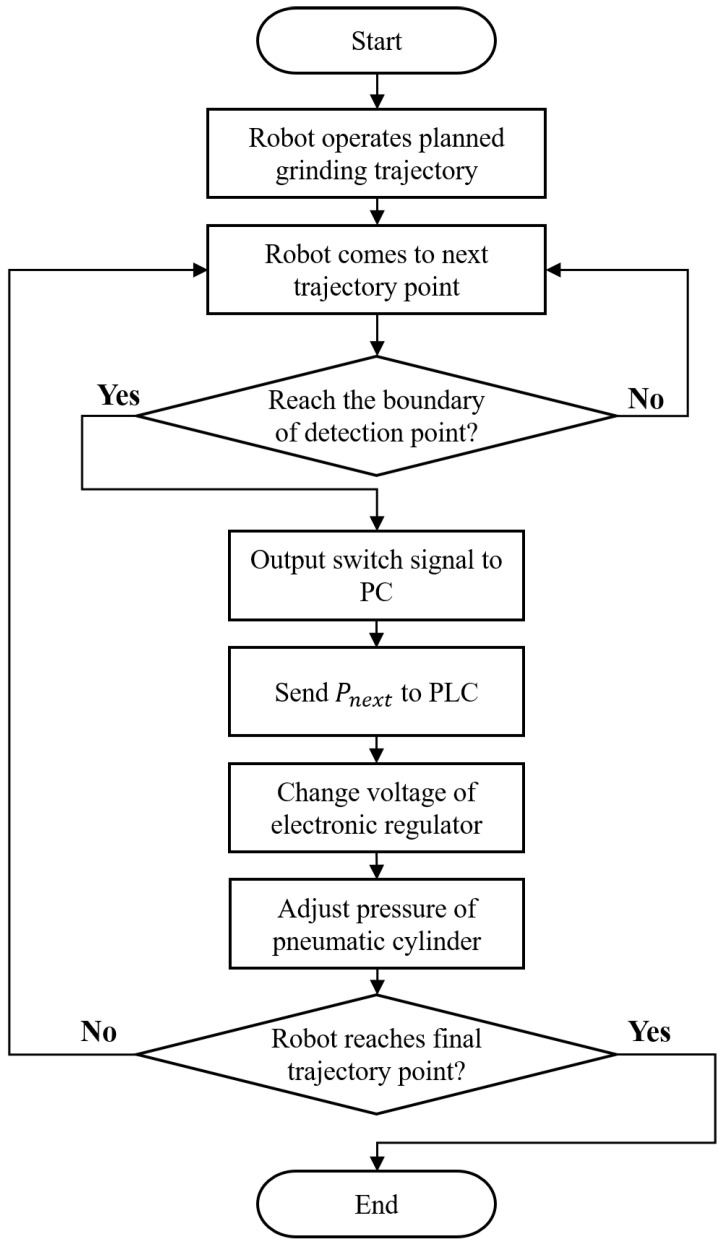
Flow chart of the grinding compensation process.

**Figure 5:**
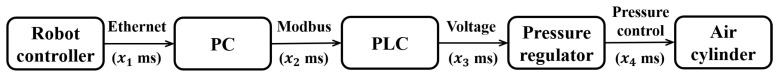
Time accumulation in multi-device communication for controlling the output pressure.

Figure 3 illustrates the flow chart of the vision sensor detection process for sensing the material residual of the workpiece at specified detection points, and the corresponding operating pressure is defined according to the determined material residual. After every operating pressure at each detection point’s corresponding area is defined and saved into a control table, the grinding compensation will be carried out by employing these calculated values to adjust the operating pressure of the pneumatic cylinder step by step. The flow chart of the grinding compensation process is shown in Figure 4. Firstly, the planned robotic grinding trajectory is conducted. The switch signals will be transmitted in turn from the robot controller to the PC when the robot reaches the boundaries of the detection points, then the defined $P_{next}$ value will be sent from the PC to the PLC to control the operating pressure. The output pressure is adjusted by the electronic regulator according to the output voltage changed using the PLC. Finally, the pneumatic cylinder’s output force $F_{c}$ is alternated immediately after the pressure is regulated by the electronic regulator. It is worth noting that the output pressure must be adjusted before the robot reaches the next trajectory point. Hence, the total operation time of changing the output pressure is considered important in multi-device communication and the robot’s motion velocity, as shown in Figure 5. The transmission time communication from the robot controller to the electronic regulator, computed by the term $x_{1}+x_{2}+x_{3}+x_{4}$, must be less than the time taken for the robot to reach the next trajectory point.

### 2.3. Compliance Grinding Analysis for the Sharpening Process

**Figure 6:**
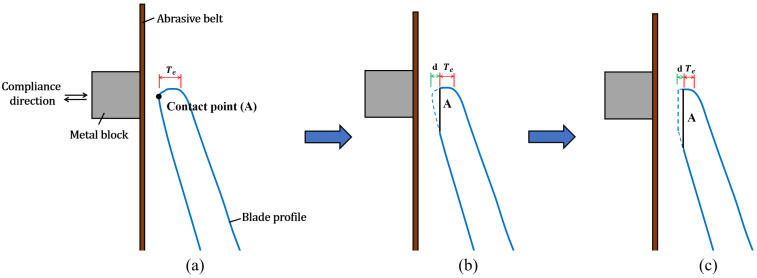
Schematic diagram of the compliant grinding compensation process. (**a**) Before initial grinding. (**b**) After initial grinding. (**c**) After grinding compensation.

Figure 6 illustrates the improved grinding compensation process for sharpening the blade-shaped workpiece with the self-designed compliance mechanism. The contact force between the workpiece and abrasive belt is passively controlled to be constant through the compliance mechanism according to the characteristics of the pneumatic cylinder referred to in Equation (1). In addition, the small constant force deviation is acceptable because the machining accuracy can be ensured with the applied iterative grinding compensations. Therefore, the effects of the small grinding force fluctuation and unpredictable abrasive belt wear are negligible. However, the stress suffered on the contact area of the workpiece is an important factor affecting the grinding performance. As shown in Figure 6a, the contact situation is close to the point contact before applying any grinding compensation. Thus, the cutting depth is relatively deeper after the initial grinding trajectory due to the higher contact stress applied to the contact area. The contact area *A* of the workpiece gradually becomes bigger after each time the grinding process is completed, as shown in Figure 6b,c. Therefore, a lower cutting depth will be made during the following grinding compensation, even if the output contact force and air pressure are kept. When the machined $T_{edge}$ is close to the required $T_{std}$, the cutting depth caused by the grinding compensation is relatively decreased due to the higher contact area. The severe over-grinding phenomena can be proven by changing the $P_{oper}$ into the lower pressure $P_{mid}$. Furthermore, the values of $P_{high}$ and $P_{mid}$ are adjusted to strengthen the weakened grinding force due to the uncontrollable abrasive belt wear to ensure the machining efficiency in long-term machining production.

## 3. Experimental Setup

**Figure 7:**
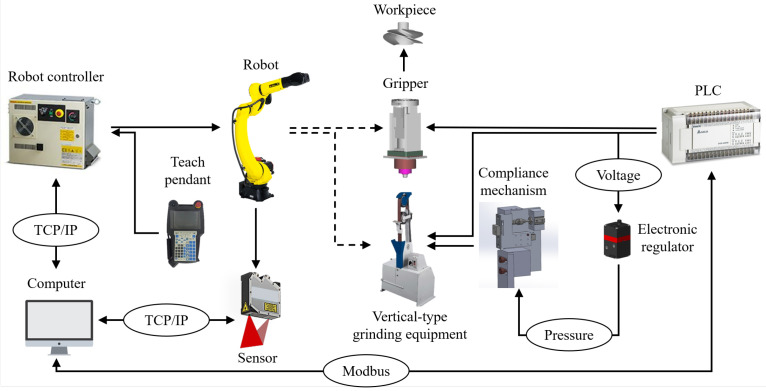
The architecture and multi-device communication of the proposed system.

**Figure 8:**
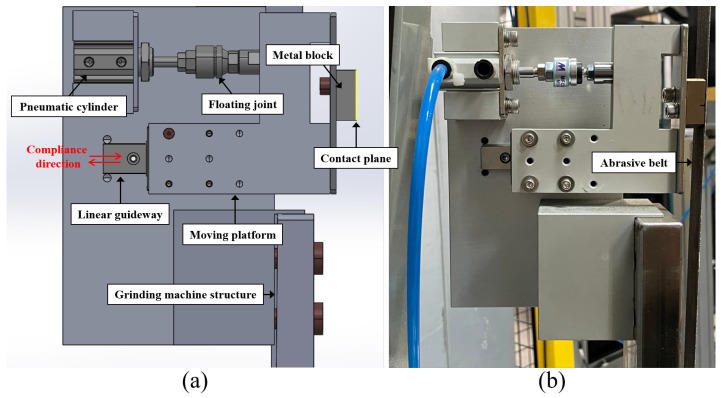
Compliance mechanism configuration. (**a**) Design prototype. (**b**) Practical installation.

In this section, to validate the functionality of the designed compliance mechanism with the proposed grinding compensation strategy, the robotic grinding workcell is established to carry out the experiments for sharpening the propeller blade. The system architecture and communication relationship between multiple devices are shown in Figure 7. In the workcell, a six-DoF industrial robot (FAUNC-M20iD/35) is employed to carry out the automatic grinding process, and a motor-driven screw-type-based gripper is installed at the end of the robot to load and unload the workpieces, which the female thread of the propeller can be screwed with the gripper. Moreover, the tested workpieces used in the experiment are annular four-blade jet ski propellers made from stainless steel. The vertical-type grinding machine is utilized in the experiment, and its available maximum velocity is 34 m/s. To acquire the workpiece’s profile data, a laser-vision sensor (LLT2960-10BL) is employed to determine the blade’s edge thicknesses. The sensing range of the laser-vision sensor in depth and width are 8 mm and 10 mm, and the corresponding data resolution are 1 μm and 8 μm, respectively. The pneumatic cylinder used in the compliance mechanism has a 10 mm stroke and a 16 mm piston diameter, and a floating joint is connected between the piston rod and the moving platform to eliminate effects of the assembly error, as shown in Figure 8. The principle design of the compliance mechanism is illustrated in Figure 8a, and the practical device configuration is demonstrated in Figure 8b. To adjust the air pressure immediately, the electronic proportional pressure regulator TECNO basic is selected to control the pneumatic cylinder because of its fast reaction time of <7 ms and considerable flow capacity of 350 L/min. The detailed specifications of the employed equipment are listed in Table 1.

## 4. Results and Discussion

**Figure 9:**
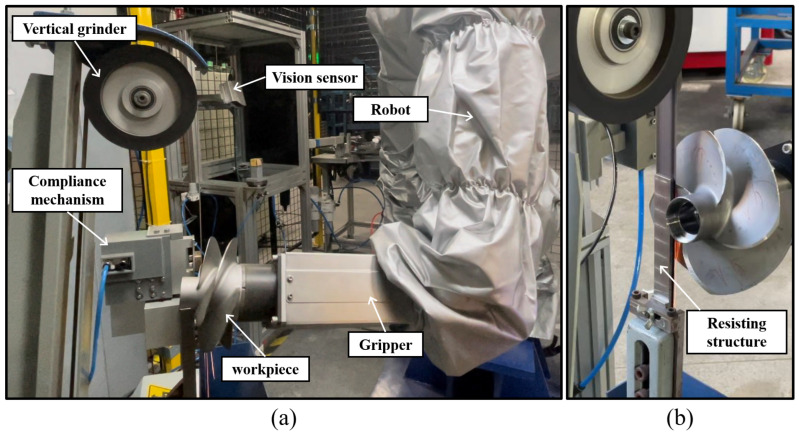
Experiment setup of the intelligent robotic grinding system for sharpening propeller blades. (**a**) With compliance mechanism. (**b**) Without compliance mechanism.

Figure 9 illustrates the experimental setup of the proposed intelligent robotic grinding system for sharpening the propeller blades. The self-designed compliance mechanism is installed into the vertical-type grinding machine, as shown in Figure 9a. The contact interface between the workpiece and abrasive belt is controlled passively to eliminate unexpected errors by adjusting the position of the pneumatic cylinder’s piston rod. To compare with the experiment setup without the compliance mechanism installed, as shown in Figure 9b, the resisting structure of the grinding machine may easily become deformed during the grinding process due to the lack of absorbing contact impact and weak structure. Hence, the grinding stability is difficult to maintain due to the unpredictable contact situation, leading to poor machining efficiency, especially in the grinding compensation process with many different longitudinal feed parameters applied [43]. To validate the practicality and reliability of the proposed compliance mechanism and control strategy, three different propeller workpiece models are employed in the experiments. In addition, numerous experimental tests were carried out to evaluate the machining efficiency compared without the compliance mechanism involved.

**Figure 10:**
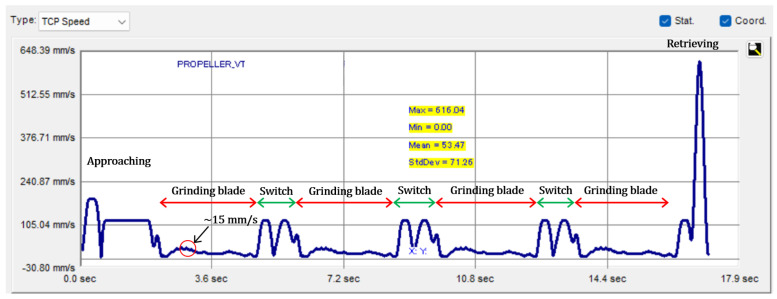
Motion analysis of the robotic grinding trajectory for sharpening a four-blade propeller.

The relative setting parameters used in the robotic grinding experiment are shown in Table 2. It is worth noting that the faster the robot feed velocity of 100 mm/s is used in the grinding experiments. However, the practical robot velocity cannot reach the setting value when the motion continuity rate is involved. In addition, the test workpieces are the four-blade propellers, with 25 trajectory points are distributed evenly on each blade edge, as shown in Figure 2a. The interval distance between any two neighbouring points is around 2.5 mm to 3.5 mm according to the size of the workpiece model. Considering the effects of the point interval and motion continuity rate, the motion analysis of a planned robotic grinding trajectory for sharpening the propeller blades is shown in Figure 10. It is apparent that the reachable maximum velocity of grinding the propeller blade is around 15 mm/s to 17 mm/s, and the velocity of 100 mm/s is reached in the switching transition of blades to save time.

Since the maximum velocity the robot can reach when grinding the propeller blade is around 17 mm/s and the minimum trajectory interval is 2.5 mm, the maximum buffer time for processing the data in the transition between the neighbouring trajectory points is computed as 147 ms. To achieve the real-time control of adjusting the pneumatic cylinder output force, the total data processing time through the robot controller to the electronic regulator must be less than 147 ms when every time the grinding compensation is conducted, otherwise the material residual cannot be removed properly. The data processing time communicating among each device is listed in Table 3. Among them, the most time-consuming part is the data transmitted from the robot controller to the PC, which is 23 ms through a Ethernet protocol. The shortest processing time to carry out one cycle of the grinding compensation is calculated as 32 ms, during which the signal is transmitted and processed from the robot controller to the electronic regulator. According to the buffer time and computed data processing time, there is enough time to change the pneumatic cylinder’s output force before the robot reaches the next trajectory point. In addition, the pneumatic cylinder and electronic regulator are connected by a 2.5 m long pneumatic tube with a 6 mm internal diameter, and the pressure range used in the experiment was around 0.4 bar to 0.7 bar. Therefore, the required flow rate to adjust the air pressure in the overall system is calculated at 44.29 L/min, assuming that the pressure is regulated within 100 ms to carry out the grinding compensation. Hence, the possibility of conducting real-time grinding compensation is proven by using the selected electronic regulator to immediately control the pneumatic cylinder because the required flow rate is much less than the capability of the electronic regulator of 350 L/min.

**Figure 11:**
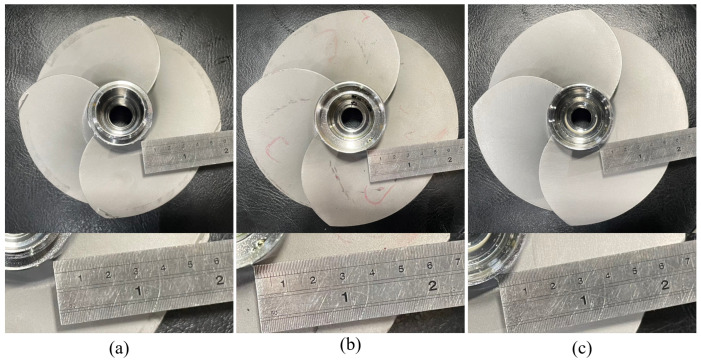
The workpiece models tested in the experiments. (**a**) Small model. (**b**) Medium model. (**c**) Large model.

To verify the reliability of the designed compliance mechanism integrated with the proposed iterative grinding compensation strategy, three different four-blade propeller models were used in the experiments, as shown in Figure 11. Comparing each of them, the models are different in size, profile, thickness, and curvature, especially the thickness distribution and curvature variation on the blade edge. The relative machining requirements of each workpiece model are illustrated in Table 4. Considering the solidness of the connection between the central structure and blades, the blade thickness at DP (1) does not need to be machined to be very sharp. In contrast, the blade thicknesses at DP (2–5) must be small than the required threshold after the grinding process is completed.

**Figure 12:**
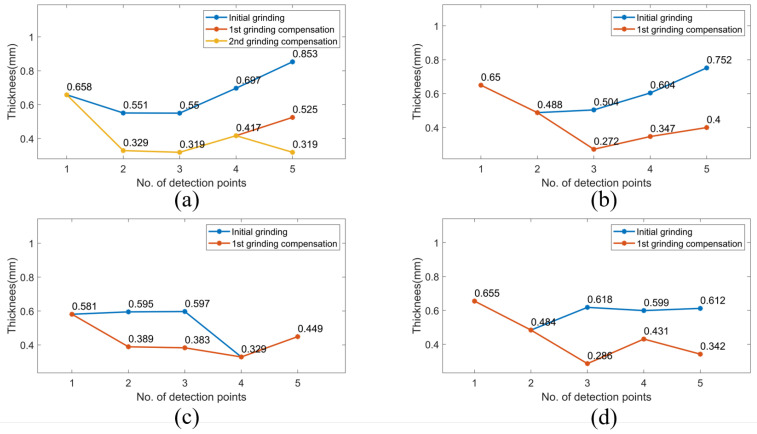
The iterative grinding compensation process for sharpening blade thickness of a small model. (**a**) blade No. 1 (**b**) blade No. 2 (**c**) blade No. 3 (**d**) blade No. 4.

**Figure 13:**
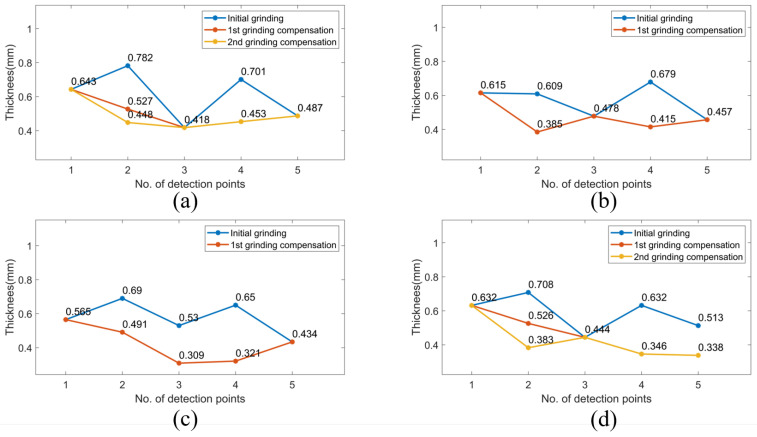
The iterative grinding compensation process for sharpening blade thickness of a medium model. (**a**) Blade No. 1. (**b**) Blade No. 2. (**c**) Blade No. 3. (**d**) Blade No. 4.

**Figure 14:**
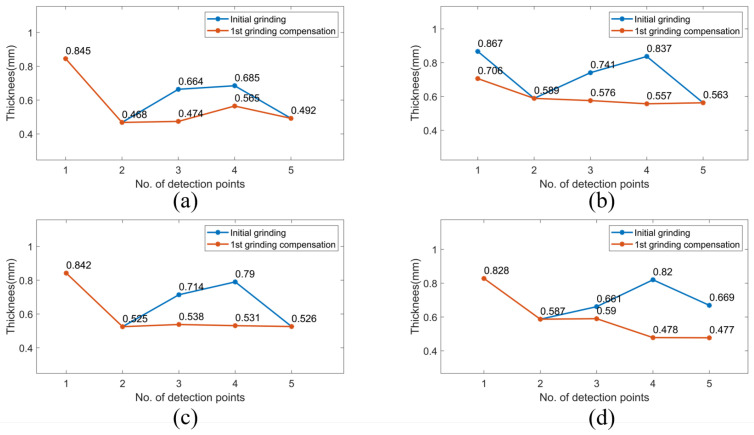
The iterative grinding compensation process for sharpening blade thickness of a large model. (**a**) blade No. 1 (**b**) blade No. 2 (**c**) blade No. 3 (**d**) blade No. 4.

**Figure 15:**
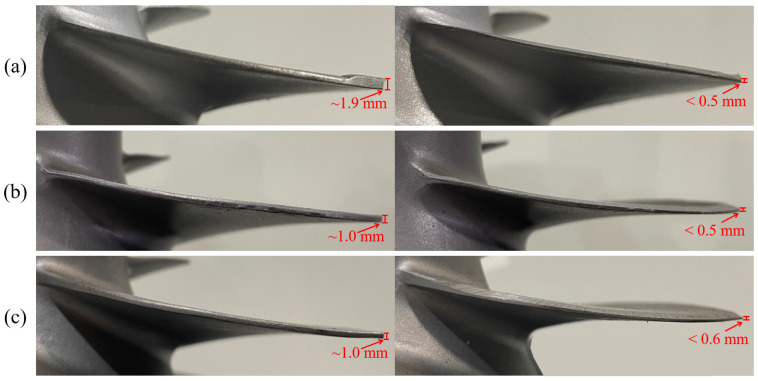
Comparison before and after the grinding process. (**a**) Small model. (**b**) Medium model. (**c**) Large model.

Figure 12, Figure 13 and Figure 14 demonstrate the blade sharpening processes of the three different propeller models: small, medium, and large, respectively. The blade thickness at each detection point was recorded by the laser-vision sensor after every time the grinding compensation process was completed. Therefore, the effectiveness of the proposed method was evaluated according to blade thickness. For instance, there are detection points on each blade that did not reach the machining threshold after the initial grinding process was finished, as shown in Figure 13. Therefore, the laser-vision sensor was used to acquire the blade profile and determine the residual material at each detection point. According to the evaluated material residuals, the operating pressure table was established to control the pneumatic cylinder’s output force during the grinding compensation process. Moreover, the operating pressure used in grinding compensation was adjusted from 0.4 to 0.7 bar according to the material residual, and the operating pressure was set at 0.1 bar to avoid material removal when the blade thickness reached the machining requirements. After the robot finished the first cycle of grinding compensation, blade No. 2 and No. 3 had already reached the machining threshold. However, there was one sub-area that failed to achieve the machining tolerances for blade No. 1 and No. 4. In the second grinding compensation, only blade No. 1 and No. 4 were scanned by the laser-vision sensor and machined by the compliance mechanism. Finally, the propeller-sharpening task was performed after the robot completed two iterations of the grinding compensation operation, each determining a blade thickness smaller than the specified machining threshold. Figure 12 and Figure 14 demonstrate the process for small and large models, finishing within two and one iterations, respectively. The comparison before and after the overall sharpening process was completed is shown in Figure 15.

**Figure 16:**
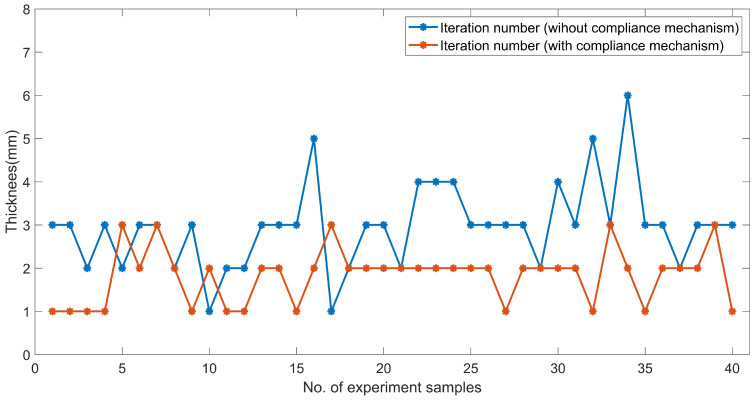
Comparison of the machining efficiency with and without the compliance mechanism.

Figure 16 compares the machining efficiency according to numerous experiments conducted with and without the compliance mechanism. The iteration number of the grinding compensation process in each experiment is recorded. In addition, the test workpiece in the experiments was the medium propeller model and the abrasive belt was not replaced. We found that the robot needed one-six iterations to complete the entire grinding process to sharpen the propeller blade without the compliance mechanism, with a maximum of six iterations. The machining efficiency was improved by introducing the compliance mechanism as several processing uncertainties were eliminated during the grinding process. For example, the workpiece could be steadily machined in the initial grinding and compensation stages because the pneumatic cylinder was employed to adaptively adjust the contact position and provide a constant force, even though the unexpected variation in the curvature of the workpiece and improper longitudinal feed parameter were involved. To compare the experiments carried out with the compliance mechanism, the robot may need one-three iterations, with a maximum of three iterations, less than without the compliance mechanism. Our study demonstrated a substantial 40% increase in the machining efficiency by implementing the compliance mechanism. This enhancement was supported by a significant difference in the average number of iterations required with and without the compliance mechanism, 1.8 and 3, respectively.

The average thickness of the medium model at each detection point was estimated at 0.525, 0.423, 0.397, 0.377 and 0.369 mm with the compliance mechanism, as shown in Table 5, with the corresponding standard deviations (SDs) of 0.096, 0.049, 0.061, 0.066, and 0.081 mm, respectively.

The average thickness and standard deviations were obtained by analysing 40 experimental findings, comprising 160 data determined at each detection point, and all measured outcomes were within the required machining thresholds. Moreover, the experimental results for the small and large models are shown in Table 5. It is worth noting that the evaluated average thickness and standard deviation with the compliance mechanism are close to the values without the compliance mechanism. Therefore, a similar grinding performance is not only achieved but demonstrated an enhanced machining efficiency when employing the compliance mechanism to integrate the proposed iterative grinding compensation strategy. Moreover, the machining stability is assured due to the smoother robotic grinding trajectory without sudden longitudinal feed changes during the grinding compensation process. Removing material in the grinding compensation stage is actively executed by the compliance mechanism.

## 5. Conclusions

This paper proposed an improved iterative grinding compensation strategy which is carried out using a self-designed compliance mechanism to enable autonomous sharpening of propeller blades even in the presence of unexpected profile deformations in the workpiece. To demonstrate the practicality of the intelligent robotic grinding system, a series of experimental trials were conducted on various workpiece models. The primary contribution of this study can be summarized as follows:

(1) A compliance mechanism is designed to work in conjunction with a vision-sensor-based iterative grinding compensation strategy, aimed at improving the performance of the robotic-based sharpening of the propeller blades. The feasibility and effectiveness of this methodology are validated through experiments, conducted on three distinct workpiece models.

(2) The relationship between the robot velocity and multi-device communication time was analysed to ensure the feasibility of the proposed grinding compensation conducted by the designed compliance mechanism.

(3) The implementation of the compliance mechanism not only ensures the attainment of the desired machining precision but also results in a significant improvement in the overall machining efficiency. The average number of iterations required to complete the propeller blade-sharpening task is reduced from 3 to 1.8, resulting in an approximate 40% improvement in overall efficiency.

(4) The compliance mechanism plays a crucial role in enhancing the stability of the machining process by preventing any structural deformation of the grinding equipment. Moreover, the effects of contact impact and vibrations are suppressed passively during the grinding process, resulting in a more reliable and consistent machining performance.

The experimental results demonstrate a significant improvement in the machining efficiency achieved by incorporating the self-designed compliance mechanism with the improved grinding compensation strategy. This approach offers a competitive advantage for high-volume production applications. It is worth mentioning that the implementation of the compliance mechanism and the improved grinding compensation strategy resulted in a noticeable improvement in machining efficiency. However, there was no significant increase observed in the machining accuracy. Therefore, the optimization of machining parameters and the enhancement of grinding equipment specifications are areas of great interest for future research aimed at improving machining accuracy.

## Figures and Tables

**Table 1:** Specifications of the experimental equipment.

Hardware	Type	Specifications
Robot	FAUNC-M20iD/35	Maximum payload: 35 kg, Working scope: 1.831 m, Repeatability accuracy: 0.02 mm
Grinding Equipment	ST-107C	Maximum velocity: 34 m/s
Grinding belt	Zirconia	Width: 2 in, Length: 96 in, Mesh number: 60
Vision sensor	LLT2960-10BL	Detection depth range: 52.5∼60.5 mm, Detection width range: −5.35∼5.35 mm, Reference resolution (Z): 1 μm Reference resolution (X): 8 μm
Electronic regulator	Tecno basic	Flow capacity: up to 350 L/min, Standard pressure range: 0∼8 bar, Reaction time: <7 ms
Pneumatic cylinder	MCMJ-11-16-10	Stroke length: 10 mm, Piston diameter: 16 mm, Floating joint: MFCS-1006T

**Table 2:** Setting parameters used in the experiment.

Parameters	Values
Robot’s feed velocity	100 mm/s
Robot’s motion continuity rate	30%
Vertical grinder speed	34 m/s
Vision sensor’s exposure, frequency, and resolution	16 ms, 62.5 Hz, 1280 points
Required flow rate	44.296 L/min
Air operating pressure	0.4 bar∼0.7 bar

**Table 3:** Data processing time communicating in each device.

Devices (Transmit)	Devices (Receive)	Processing Time (ms)
Robot controller	PC	23
PC	PLC	1.4
PLC	Electronic regulator	<1
Electronic regulator	Pneumatic cylinder	7

**Table 4:** Machining requirements of the different workpiece models.

Threshold (mm)	DP (1)	DP (2)	DP (3)	DP (4)	DP (5)
Small model	0.85	0.5	0.5	0.5	0.5
Medium model	0.7	0.5	0.5	0.5	0.5
Large model	0.85	0.6	0.6	0.6	0.6

**Table 5:** Comparison of the average thickness and standard deviation with and without the compliance mechanism.

Thickness (mm)	Model: Small (With compliance mechanism)				
	DP (1)	DP (2)	DP (3)	DP (4)	DP (5)
Average	0.706	0.399	0.407	0.404	0.403
SD	0.078	0.050	0.056	0.051	0.057
	Model: Medium (With compliance mechanism)				
	DP (1)	DP (2)	DP (3)	DP (4)	DP (5)
Average	0.525	0.423	0.397	0.377	0.369
SD	0.096	0.049	0.061	0.066	0.081
	Model: Large (With compliance mechanism)				
	DP (1)	DP (2)	DP (3)	DP (4)	DP (5)
Average	0.804	0.544	0.547	0.555	0.540
SD	0.043	0.036	0.035	0.046	0.048
	Model: Medium (Without compliance mechanism) [43]				
	DP (1)	DP (2)	DP (3)	DP (4)	DP (5)
Average	0.528	0.393	0.393	0.385	0.345
SD	0.07	0.041	0.036	0.045	0.082

## Data Availability

The data presented in this study are available on request from the corresponding author. The data are not publicly available due to privacy.
